# Intussusception and Gut Dysmotility: A Systematic Review Assessing Unexpected Complications of Bariatric Surgery

**DOI:** 10.7759/cureus.58086

**Published:** 2024-04-11

**Authors:** Kainaat Shergill, Kusalik Boppana, Naiela E Almansouri, Saloni Bakkannavar, Youmna Faheem, Amisha Jaiswal, Samia Rauf R Butt

**Affiliations:** 1 Department of General Surgery, California Institute of Behavioral Neurosciences & Psychology, Fairfield, USA; 2 Medicine, Maharishi Markandeshwar Institute of Medical Sciences and Research, Mullana, IND; 3 Department of Internal Medicine, California Institute of Behavioral Neurosciences & Psychology, Fairfield, USA; 4 Department of Internal Medicine, University of Tripoli, Tripoli, LBY; 5 Department of Pediatrics, California Institute of Behavioral Neurosciences & Psychology, Fairfield, USA

**Keywords:** bariatric surgery mesh, bariatric surgery, weight-loss intervention, bariatric and endocrine surgery, bariatric surgery complications, retrograde intussusception, small bowel intussusception, jejunal intussusception

## Abstract

Bariatric surgery, although effective in treating obesity-related comorbidities, rarely results in intussusception, which is a severe complication. This study aimed to enhance clinical practice and establish early diagnosis by elucidating risk factors and management strategies associated with intussusception. We conducted this systematic review following Preferred Reporting Items for Systematic Reviews and Meta-Analysis 2020 criteria. We looked through PubMed, PubMed Central, ScienceDirect, ScienceOpen, MyScienceWork, Hyper Articles en Ligne (HAL), Google Scholar, and the Medical Literature Analysis and Retrieval System Online for relevant studies and research. Articles were screened according to inclusion and exclusion criteria, and relevance. We employed pertinent quality appraisal instruments to look for bias. Initially, we discovered 2,833 items. We eliminated redundant and unnecessary publications. After reviewing all the articles, we selected 30 studies based on their titles and abstracts. Out of the 30 studies reviewed, 12 papers were included in this review, with the remaining 18 being eliminated due to low quality. Medical practitioners and surgeons have a responsibility to meticulously monitor and provide postoperative surveillance, with a particular emphasis placed on individuals exhibiting symptoms of abdominal pain and vomiting, as there is a clinical imperative to consider the possibility of intussusception. The management approach, whether conservative or surgical, remains contingent upon the clinical context.

## Introduction and background

Severe obesity, defined as a body mass index (BMI) of ≥40 kg/m^2^, has not only emerged as an important clinical problem but has also rapidly become a preoccupying public health issue [1]. The obesity epidemic has reached unprecedented levels globally. In tandem with this surge, the demand for bariatric surgery (BS) has risen substantially. The rise in demand for BS reflects not only the escalating prevalence of obesity but also a strategic response to address associated comorbidities, such as diabetes and hypertension. Surgery is the most effective long-term treatment for severe obesity. It improves the quality of life and reduces long-term morbidity and mortality [2]. The laparoscopic Roux-en-Y gastric bypass (LRYGB) is currently one of the most commonly performed bariatric procedures to treat morbid obesity [3]. However, BS is not free of complications. A less common yet potentially catastrophic complication of Roux-en-Y gastric bypass (RYGB) is intussusception [4]. Retrograde intussusception (RI) after RYGB surgery occurs at the jejunojejunostomy (JJ) almost exclusively, with an antiperistaltic (retrograde) telescoping of the common limb into the jejunal anastomosis. Such cases differ from traditional intussusception cases due to the antegrade process [5]. Typically, it presents late after open or laparoscopic procedures with intermittent partial or complete bowel obstruction. It can be challenging to diagnose intussusception by preoperative clinical diagnosis because patients can present with a range of symptoms. Abdominal pain may be acute, chronic, or intermittent, and obstructive symptoms may vary in intensity depending on the degree of obstruction [6].

The pathophysiology behind the retrograde nature observed in patients remains unclear. Several theories have been suggested, but the most frequently recognized one currently links a motility issue to the onset of intussusception. There is insufficient knowledge about the exact mechanism that causes intussusception in patients undergoing RYGB surgery. Theories suggest that the jejunojejunal anastomosis could serve as a focal point or that asynchronous peristaltic movements originating from the Roux limb could cause the bowel to invaginate onto itself. Serious consequences include intestinal ischemia, necrosis, and bowel blockage that might result from small intestine intussusception [7,8]. It is more commonly seen in females who experience rapid weight loss. Women who are expecting a child and have had BS in the past should be treated as high-risk patients and given extra care. Pregnancy may increase the risk of surgical problems from prior BS due to several factors, such as hyperemesis, an increase in uterine volume, and elevated abdominal pressure [9]. There is disagreement about whether a reduction is adequate or whether an enteropexy, resection, or reconstruction of the JJ anastomosis needs to be done when it comes to surgical treatment. There is not a set treatment strategy in place. Before surgery, intussusceptions may spontaneously resolve, but they can also occasionally result in intestinal blockage or ischemia. If the small bowel is viable, treatment options may be restricted to reduction; however, resection of the afflicted segment appears to reduce the likelihood of recurrence.

Since the existing literature on this topic is limited, the pathophysiology of the disease remains unclear. Most often presenting with a missed diagnosis, the disease can have a wide range of clinical presentations and can present years after the patient has undergone surgery. By synthesizing the existing literature on BS, this review aims to assess the risk of gut dysmotility, intussusception, and subsequent obstruction in patients undergoing BS, its predictive factors, management, and subsequent operative treatment options to provide a consensus on management options to help in easy and early identification of the disease. With the increasing popularity of BS, the number of patients undergoing these procedures will likely increase in subsequent years, and this information may prove critical to guiding clinical practice.

## Review

Methods

We rigorously adhered to the Preferred Reporting Items for Systematic Reviews and Meta-Analysis (PRISMA) guidelines to maintain transparency, thoroughness, and methodological accuracy in our study. Our comprehensive literature review, facilitated by PRISMA methodology, ensured the inclusion of all relevant studies using carefully formulated search terms. We selected studies based on clear inclusion/exclusion criteria, addressing various inquiries. Following PRISMA guidelines, we chose papers meeting requirements after preliminary assessments of titles, abstracts, and full text [10].

Database Search Protocol

This comprehensive review was conducted using PubMed, PubMed Central, Medical Literature Analysis and Retrieval System Online, ScienceDirect, ScienceOpen, MyScienceWork, Hyper Articles en Ligne (HAL), and Google Scholar as research databases and search engines. The study utilized the methods of BS, intussusception, and gastrointestinal dysmotility. As indicated in Table 1, we combined the pertinent concepts with particular keywords using the Boolean term "OR."

**Table 1:** PubMed search strategy with regular keywords

Concepts	Keywords	PubMed search builder
Bariatric surgery	Gastric bypass or weight loss surgery	Gastric bypass or weight loss surgery
Intussusception	Intussusception or gut dysmotility	Intussusception or gut dysmotility

Similarly, a Medical Subject Headings (MeSH) strategy was created using the same concepts as keywords. Subheadings such as complications, adverse effects, and surgery were chosen. The findings are shown in Table 2. A similar method was used for the advanced search strategy, as showcased in Table 3.

**Table 2:** MeSH strategy MeSH: Medical Subject Headings

Keywords	MeSH strategy
Bariatric Surgery	(("Bariatric Surgery/adverse effects"[Majr] OR "Bariatric Surgery/classification"[Majr] OR "Bariatric Surgery/rehabilitation"[Majr] OR "Bariatric Surgery/statistics and numerical data"[Majr]))
Intussusception	("Intussusception/pathology"[Majr] OR "Intussusception/physiopathology"[Majr] OR "Intussusception/prevention and control"[Majr] OR "Intussusception/surgery"[Majr] OR "Intussusception/therapy"[Majr])

**Table 3:** Advanced search strategy

Advanced search strategy
(("Bariatric Surgery/adverse effects"[Majr] OR "Bariatric Surgery/classification"[Majr] OR "Bariatric Surgery/rehabilitation"[Majr] OR "Bariatric Surgery/statistics and numerical data"[Majr])) AND ("Intussusception/pathology"[Majr] OR "Intussusception/physiopathology"[Majr] OR "Intussusception/prevention and control"[Majr] OR "Intussusception/surgery"[Majr] OR "Intussusception/therapy"[Majr])

Screening of Articles

All the relevant articles were collected, and duplicates were eliminated. The appropriate articles were then filtered by full-text, title, and abstract reading. After that, 12 research papers were chosen and put through a quality assessment process.

Inclusion Criteria

This paper focused on the following studies: studies involving adult patients (18 years or older) who had undergone any BS (gastric bypass, sleeve gastrectomy, and adjustable gastric banding); primary research studies, including randomized controlled trials, cohort studies, case-control studies, and observational studies published in the last six years as full-text articles in English; studies that reported on the incidence and risk, and investigated and identified predictive factors or risk factors associated with gut dysmotility, intussusception, and subsequent obstruction after BS and studies discussing management strategies and surgical or operative treatment options for patients with intussusception and obstruction following BS.

Exclusion Criteria

The exclusion criteria included the following: studies involving pediatric patients (under 18 years old) and geriatric patients (above 65 years old); studies not related to BS or focused on other types of surgery; studies conducted on animals rather than human subjects; studies published in languages other than English unless English translations were available; studies with incomplete or insufficient data regarding the outcomes, predictive factors, management, or operative treatment and studies published before the year 2018 and studies based on the gray literature.

Quality Assessment

This systematic review included case studies, observational studies, systematic reviews, and meta-analyses. Quality appraisal tools such as the Joanna Briggs Institute (JBI) checklist tool, the Newcastle-Ottawa Scale, and AMSTAR (A MeaSurement Tool to Assess systematic Reviews) checklist were applied to assess the risk of bias during the paper selection process. Only articles that satisfied above 70% of the requirements were selected. Tables 4, 5 display the caliber of the chosen articles.

**Table 4:** Quality assessment of the included case reports and cohort study JBI tool: Joanna Briggs Institute tool JBI Critical Appraisal Checklist 1. Were the patient’s demographic characteristics clearly described? 2. Was the patient’s history clearly described and presented as a timeline? 3. Was the current clinical condition of the patient on presentation clearly described? 4. Were diagnostic tests or assessment methods and the results clearly described? 5. Was the intervention(s) or treatment procedure(s) clearly described? 6. Was the postintervention clinical condition clearly described? 7. Were adverse events (harms) or unanticipated events identified and described? 8. Does the case report provide takeaway lessons? Newcastle-Ottawa Cohort Checklist 1. Representativeness of the exposed cohort. 2. Selection of the nonexposed cohort. 3. Ascertainment of exposure. 4. Demonstration that the outcome of interest was not present at the start of the study. 5. Comparability of cohorts on the basis of the design or analysis: a—study controls for the most important factor, b—study controls, or any additional factor. 6. Assessment of outcome. 7. Was the follow-up long enough for outcomes to occur? 8. Adequacy of follow-up of cohorts.

Author	Type of study	Quality appraisal tool	1	2	3	4	5	6	7	8
Doño et al. [11]	Case report	JBI	+	+	-	+	+	?	+	+
Bhadra et al. [12]	Case report	JBI	+	-	?	+	+	+	+	+
Chys et al. [13]	Case report	JBI	+	+	+	?	+	+	+	+
Michiels et al. [14]	Case report	JBI	+	+	-	+	+	+	+	+
Teixeira et al. [15]	Case report	JBI	+	+	+	?	+	-	+	+
Elkbuli et al. [16]	Case report	JBI	+	+	?	+	+	+	+	+
Machado et al. [17]	Case report	JBI	+	+	-	+	+	+	+	+
Facchiano et al. [18]	Case report	JBI	+	+	+	+	+	+	+	+
Zaigham et al. [19]	Cohort	The Newcastle-Ottawa Scale	+	+	+	+	+	+	+	+

**Table 5:** Quality assessment of included systematic reviews and meta-analysis AMSTAR: A MeaSurement Tool to Assess systematic Reviews AMSTAR Checklist 1. Was an "a priori" design provided? 2. Was there duplicate study selection and data extraction? 3. Was the comprehensive literature search performed? 4. Was the status of publication (i.e., gray literature) used as inclusion criteria? 5. Was the list of studies (included and excluded) provided? 6. Were the characteristics of the included studies provided? 7. Was the scientific quality of the included studies assessed and documented? 8. Was the scientific quality of the included studies used appropriately in formulating a conclusion? 9. Were the methods used to combine the findings of studies appropriate? 10. Was the likelihood of publication bias assessed? 11. Was the conflict of interest stated?

Author	Type of study	Quality appraisal tool	1	2	3	4	5	6	7	8	9	10	11
Bonouvrie et al. [20]	Systematic review	AMSTAR checklist	+	+	+	+	+	+	+	+	?	+	+
Oor et al. [21]	Meta-analysis	AMSTAR checklist	+	+	+	+	+	+	+	+	?	?	+
Petrucciani et al. [22]	Systematic review	AMSTAR checklist	+	+	?	+	+	+	+	+	+	+	+

Results

**Figure 1:**
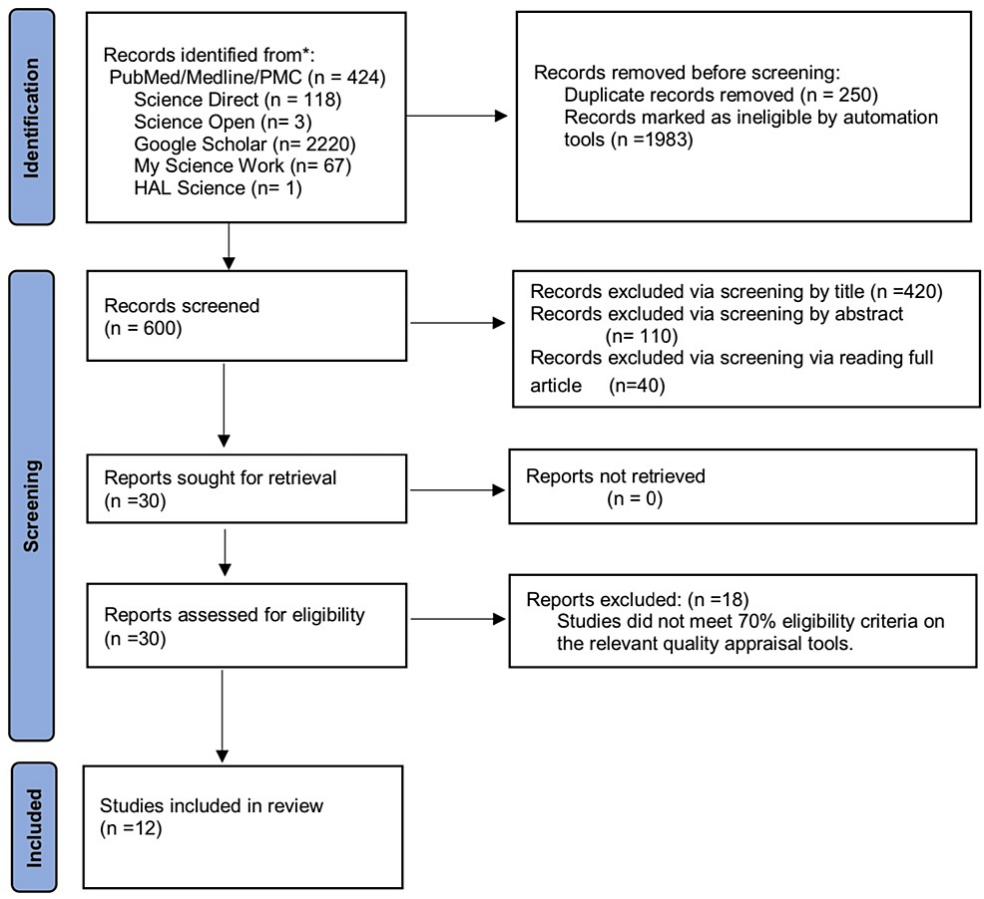
PRISMA flowchart PRISMA: Preferred Reporting Items for Systematic Reviews and Meta-Analysis; PMC: PubMed Central *p < 0.05

To find suitable studies, we electronically searched six databases. At first, we discovered 2,833 articles pertaining to our subject. Automation tools then eliminated 1,983 documents and 250 duplicates due to ineligibility. After screening according to inclusion/exclusion criteria and considering pertinent title, abstract, and full-text reading, this number was further reduced to 30. Finally, the quality assessment tools evaluated the bias in the research. Ultimately, we eliminated the lower quality articles and finalized 12 studies. The search approach utilized to carry out this review is shown in a PRISMA flowchart in Figure 1 [10].

The summary of the finalized articles is displayed in Tables 6, 7.

**Table 6:** Summary of included case reports and case series RYGB: Roux-en-Y gastric bypass; JJ: jejunojejunal

Author and year of publication	Type of study	Patient demographics	Past history	Presentation	Management	Conclusion
Doño et al., 2022 [11]	Case report	63-year-old female	Surgical history included two cesarean sections, laparoscopic cholecystectomy, and an open RYGB performed 32 years ago	24- to 48-hour history of stomach pain radiating to the left flank from the left lower quadrant that became worse	The dilated Roux and biliopancreatic limbs were reanastomosed to the distal common channel	It is especially appropriate to treat post-RYGB individuals who experience persistent, sporadic, or colicky abdomen pain with a high degree of clinical suspicion
Bhadra et al., 2018 [12]	Case report	40-year-old female	Surgical history of laparoscopic gastric bypass with Roux-en-Y anastomosis in 2009	Worsening abdominal pain, 10/10 in intensity, and tearing in character associated with nausea and vomiting frank blood	Resection and anastomosis with perforation repair	Any delay in timely intervention can increase fetomaternal mortality to a large extent. Surgery within 48 hours has 10% mortality, whereas, after 48 hours, it is 50%
Chys et al., 2020 [13]	Case series	56-year-old female and 58-year-old male	Female: 11 years ago, an RYGB surgery was performed. Male: 9 years ago, an RYGB surgery was performed	Female: she suddenly had significant left subcostal pain overnight. Male: intense, sudden discomfort in the abdomen	Female: a stapled Barcelona side-to-side repair was used after a common limb segmentectomy. Male: a JJ anastomosis enterotomy was carried out. JJ anastomosis was disassembled and reconstructed	One possible explanation might be ectopic pacemakers causing retrograde peristalsis. The best course of action seems to be a segmentectomy
Michiels et al., 2019 [14]	Case report	33-year-old female	Surgical history of gastric banding and a laparoscopic RYGB performed in 2015 for class III obesity	Acute abdominal pain without nausea or vomiting	Resection of the blind extremity of the biliopancreatic limb	After RYGB, small bowel intussusception is an uncommon long-term consequence. If this uncommon ailment is not identified and treated immediately, it may induce blockage and result in intestinal necrosis
Teixeira et al., 2020 [15]	Case report	42-year-old female	Surgical history of laparoscopic RYGB two years ago	Stomach distension, nausea, vomiting, and irregular bowel and flatus	The jejunojejunostomy was removed together with the ischemic bowel (common limb). In order to redo the RYGB, the latter was rebuilt using two lateral jejunojejunal anastomoses	To prevent intestinal necrosis, an early surgical intervention would have been ideal. Therefore, a high level of suspicion is necessary, especially when postbariatric surgery is involved and clinical manifestations of intra-abdominal emergencies may be less flamboyant
Elkbuli et al., 2020 [16]	Case report	22-year-old female	Surgical history of laparoscopic RYGB six years ago	12-hour history of abdominal pain	Adhesion lysis, internal hernia reduction, untwisting of jejunojejunal anastomosis, reduction of jejunojejunal anastomosis intussusception, and closure of the jejunojejunal space	An unusual complication of RYGB involving intussusception, internal hernia, and volvulus that was successfully managed without the need for bowel resection due to early identification and surgical intervention
Machado et al., 2018 [17]	Case report	54-year-old female	Surgical history of a gastric bypass six years ago	Acute onset of abdominal pain, nausea, and nonbilious vomiting	Reduction of the intussuscepted 60 cm of small bowel via laparoscopy	After RYGB, retrograde intussusception is an uncommon emergency situation. To avoid mesenteric ischemia and necrosis, early diagnosis is crucial
Facchiano et al., 2018 [18]	Case report	38-year-old female	Surgical history of RYGB five months ago	Acute abdominal pain, alimentary and bilious vomiting, and fever	Resection of the jejunojejunal anastomosis	A laparoscopic approach to treat bowel intussusception after RYGB is safe and feasible

**Table 7:** Summary of included observational studies and systematic reviews SBO: small bowel obstruction; RYGB: Roux-en-Y gastric bypass; CT: computed tomography; JJ: jejunojejunostomy

Author and year of publication	Purpose of study	Number of patients	Type of study	Result	Conclusion
Zaigham et al., 2023 [19]	To correlate radiological findings with clinical outcomes to differentiate intussusceptions requiring emergency surgery for SBO	35	Cohort	Proximal bowel dilatation predicted SBOs of any cause as well as SBO caused by an intussusception	Intussusception length >100 mm on CT in RYGB patients is an easy and valuable sign indicating SBO that may require emergency surgery
Bonouvrie et al., [20]	To identify characteristics of intussusception in pregnant women who have undergone a laparoscopic RYGB	23	Systematic review	Of 23 intussusceptions, 17 were retrograde, with the majority (18/23) occurring at the jejunojejunostomy. Only manual reduction proved effective in treating six cases, while surgical excision was necessary for 17 others. Of the patients, 15 (65%) had an ischemic segment. Six (26%) of the patients gave birth while they were hospitalized. There was one documented fetal death (of twins)	As a serious consequence that increases the risk of maternal morbidity, newborn morbidity, and mortality, prompt diagnosis and surgical resection are necessary if nonrecoverable ischemia or necrosis is discovered
Oor et al., 2021 [21]	This study aimed to determine the incidence of intussusception following RYGB and provide insight into outcomes of subsequent operative treatment	24,655	Systematic review	With no anastomotic leakages reported by any of the retrospective investigations, the benefit of resection in terms of potentially lower recurrence rates is further reinforced by the low reported morbidity rates associated with resection. More importantly, the authors feel there should be a very low threshold for resection and subsequent revision of the JJ because of the high prevalence of ischemia described in the included studies	A negative CT scan cannot completely rule out intussusception, and early surgical exploration is necessary because of the high risk of ischemia. Since ischemia is common, resection and subsequent revision of the JJ should be done at a low threshold. Reduction alone seems to be linked to a high recurrence rate
Petrucciani et al., 2020 [22]	To analyze the clinical presentation, diagnostic procedures, and treatment of surgical complications of bariatric surgery during pregnancies	70	Systematic review	Six of the 10 intussusception patients required intestinal resection, while the remaining four instances only required intussusception reduction	Pregnancy-related surgical complications from prior bariatric surgery constitute a difficult and intricate clinical entity with potentially serious consequences. In cases when pregnant women have a high clinical and radiologic suspicion of a surgical complication from prior bariatric surgery, prompt surgical exploration is required

Discussion

Predisposing Factors

The studies examined diverse post-BS timelines, highlighting instances where patients presenting with complications such as intussusception, motility issues, and intestinal obstruction underwent surgery over varying intervals, ranging from several months to multiple years. Doño et al. reported a case where a patient presented with symptoms 32 years after undergoing open RYGB [11]. Bhadra et al. reported symptoms emerging nine years after surgery, while Chys et al. indicated a variation of 9-11 years between presentation and surgery [12,13]. Conversely, Michiels et al. presented a case with a comparatively shorter gap of two years between surgery and symptom manifestation [14]. Teixeira et al. observed symptoms emerging two years after BS, whereas Elkbuli et al. and Machado et al. showcased patients developing symptoms six years after their respective surgeries [15-17]. Facchiano et al. presented a case in which symptoms manifested five months after surgery [18]. In the investigation conducted by Zaigham et al. in 2023, findings indicated that among a cohort of 35 individuals presenting with acute symptoms, nine patients required emergency surgical intervention within 24 hours. This study highlighted the imperative to refrain from trivializing the condition, as its apparent clinical gravity was substantiated by the observed need for expeditious surgical measures, signifying the potential for fatal consequences [19].

Contrary to the prevailing characterization of intussusception as an exceptionally infrequent occurrence, Bonouvrie et al. challenged this perception by demonstrating its presence in a notable proportion. Specifically, Bonouvrie et al. illustrated instances of intussusception in up to 7% of cases involving small bowel obstruction (SBO) after LRYGB. Moreover, the author posited that the actual incidence may surpass anticipated rates, deviating from prior literature expectations [20]. According to Oor et al., the St. Antonius Hospital in Nieuwegein, The Netherlands, has an average of 800 BSs performed per year, of which about 500 are RYGB procedures and 300 are sleeve gastrectomies. According to their experience, women made up about 75% of patients receiving primary RYGB surgery. This proportion is similar to what other authors have documented and helped explain why most post-RYGB intussusception cases are women [21]. The evaluation by Petrucciani et al. included 120 instances of pregnant women who had emergency surgery due to complications from a prior bariatric procedure. The characteristics of this population were analyzed, along with the diagnosis, course of treatment, and results. Ten of these pregnant patients developed intussusception, and surgical intervention was needed in six cases [22]. These observations underscored the need to reconsider the perceived rarity of intussusception in the context of postoperative complications following LRYGB procedures. Clinicians must be aware of the potential associated with obstructive complications and maintain a low threshold of suspicion in these cases [23].

The most susceptible seemed to be women who had successful weight loss [13,16]. The woman in Michiels et al.'s study had RYGB for class III obesity in 2015; in the two years following the procedure, she had lost more than 50 kg, and her BMI had dropped from 43.7 to 25.1 kg/m^2^ [14]. According to Machado et al., a female patient presented with considerable weight loss six years after the RYGB, with an excess weight loss of 64.7% [17]. Intussusception was typically observed in female patients who had undergone significant weight reduction, according to Doño et al. [11]. Another theory was that the bypassed jejunum's fixed mesentery became hypermobile due to mesenteric fat loss [20]. Facchiano et al. stated that weight loss contributes to intussusception because of the lower resistance related to the decreased thickness of the mesentery of the intussuscepted segment [18]. Doño et al. reported a patient with a recorded 48% weight loss after RYGB, which is consistent with the finding that patients with an interval drop in BMI of 15.8 kg/m^2^ are at an increased risk of developing RI [11]. In consensus with the selected studies, rapid weight loss, female gender, and pregnancy may constitute predisposing factors for the development of intussusception. It is not clear whether pregnancy increases the risk of intussusception, but it may act as a predisposing factor.

Etiology and Pathogenesis

The pathophysiological aspects behind the cause of the condition largely remain unclear and multiple theories have been suggested. According to Machado et al., any ailment that interferes with the bowel's regular peristalsis increases the risk of intussusception [17]. An underlying motility issue is presently the most widely accepted explanation and is thought to be a major cause of intussusception [11,15]. This theory, according to Chys et al., is the foundation for the Roux stasis syndrome, a condition that is commonly brought on by eating and manifests as poor stomach emptying, discomfort, nausea, and vomiting [13]. According to Facchiano et al., even though this phenomenon does not explain the incidence of retrograde antiperistaltic intussusception, it has been hypothesized that the suture line at the JJ could operate as a lead point [18]. Disrupted bowel motility is also suggested to be the cause by Chys et al. and Zaigham et al. [13,19]. In contrast, in non-RYGB adult patients, a lead point such as a tumor or diverticulum may be present, which may cause the bowel to telescope most frequently in an anterograde fashion. In post-RYGB patients diagnosed with intussusception, no lead point is identified during surgical exploration, and all intussusceptions involve the jejunojejunostomy site, more often in a retrograde fashion and occasionally in an anterograde fashion [21]. Elkbuli et al. and Doño et al. also stated that intussusception most commonly occurs at the jejunojejunostomy site in a retrograde fashion in post-RYGB patients [11,16]. Most intussusceptions are retrograde but can be anterograde. In patients who are not pregnant, RI is more common [24].

The duodenum is cut off from the food passage when the jejunum is cut open and placed back on the gastric pouch. The duodenum has the highest capacity to set pacesetters because of its fast-paced nature. When the Roux-en-Y structure forms, the pacesetting triggered by food passage is lost, allowing ectopic pacemaker potentials to occur within the Roux limb. Retrograde peristalsis is made possible by the ectopic potentials colliding at the JJ site and being able to move in an oral direction. In surgically transecting and reanastomosing the jejunum, the natural peristaltic rhythm initiated by the duodenum, particularly in response to food passage, is bypassed. This bypassing results in the development of abnormal pacemaker sites within the jejunum, which can disrupt normal intestinal motility because of the foci of ectopic pacemakers along the jejunum, increasing the potential for RI [6]. Bowel dysmotility has been hypothesized as a leading cause [25]. According to Bonouvrie et al., the lead point with an antegrade intussusception, proximal to distal orientation, is the basis for the etiology in the general population. The most widely recognized explanation for the emergence of RI following LRYGB surgery is the creation of ectopic pacemakers, which are essential in generating an unstable zone with reverse peristalsis [20]. According to the study by Oor et al., ectopic pacemakers may arise in the Roux limb during the construction of the Roux limb and, consequently, transection of the jejunum, where small bowel motility is initiated. This can result in both anterograde and retrograde peristalses, with the JJ acting as a meeting point, or lead point, where peristaltic waves from the duodenum encounter ectopic waves from the Roux limb, leading to intussusception [21,26]. Initially, it was thought that the anastomotic staple line acted as a leading point because of scarring, but it was postulated that irregularities in the pacesetters distal to the JJ anastomosis could result in atony and distension of the Roux limb and associated antiperistaltic movement [27]. Some studies also suggest that the iatrogenic lead point created by the suture or staple line at the enteroenteral anastomosis from the previous surgery may generate hyperperistalsis of the excluded segment, causing the biliopancreatic limb to telescope into the common limb [28,29].

In pregnancy, the available evidence suggests the absence of an elevated risk of adverse perinatal or obstetrical outcomes, encompassing congenital anomalies, even in pregnancies occurring during the phase of maximal weight loss following BS. When juxtaposed with pregnancies antedating bariatric interventions, those subsequent to such procedures exhibit diminished incidences of hypertension and diabetes, reduced maternal weight gain, and comparable average birth weights. A review of nine pregnancy-related intestinal obstructions after surgery revealed that the nonspecific nature of abdominal complaints early in the course of RYGB-associated obstruction might incorrectly be attributed to common obstetrical complaints such as morning sickness, hyperemesis, reflux, and uterine contractions, which may delay prompt diagnosis and treatment in pregnant patients [30]. Potential complications of the procedure should be routinely considered in pregnant and nonpregnant patients who present with abdominal pain [31].

Clinical Presentation

Most of the patients in the included studies presented with vague symptoms that make establishing the diagnosis challenging and difficult. The symptoms might not be able to be distinguished from other frequently occurring side effects such as internal hernias or marginal ulcers [11]. For cases that have been documented, the median period at presentation is 36 weeks after surgery [2]. The studies by Chys et al., Michiels et al., and Elkbuli et al. reported that the patient just presented with abdominal pain solely [13,14,16]. Doño et al. reported that the patient presented with worsening abdominal pain with pain radiation to the flank: 10/10 on the pain scale associated with nausea and constipation [11]. Bhadra et al.'s report showcased a patient with 10/10 worsening abdominal pain with nausea and episodes of frank blood vomiting [12]. According to Teixeira et al., the patient reported having nausea, vomiting, intermittent stomach pain, distension of the abdomen, and no feces or flatus [15]. Machado et al. reported a patient with symptoms of abdominal pain and nonbilious vomiting, whereas Facchiano et al. reported a patient with abdominal pain, bilious vomiting, and fever [17,18]. Thus, the patients might present with diverse and varied symptoms, and carrying out a prompt diagnosis based on symptoms alone may prove unfruitful. Patients might have other superimposed or underlying conditions such as lead points or intestinal obstruction and bowel ischemia. Very few cases of acute SBO in an RYGB-complicating pregnancy have been described in the medical literature [26,27].

According to the studies that were chosen, patients typically present with severe stomach pain, nausea, and vomiting. However, the degree of intussusception affects the length and severity of symptoms. While total intussusception with small intestinal blockage and strangulation may result in a more acute and severe presentation, partial and intermittent intussusception may induce mild and intermittent, occasionally chronic problems. According to Oor et al., one could even speculate that intussusception could be the reason for sporadic abdominal pain in the difficult subset of post-RYGB patients experiencing "unexplained" abdominal pain. Blood test results that reveal leukocytosis, high C-reactive protein levels, elevated pancreatic enzymes, hematemesis, bloody diarrhea, peritonitis, or a palpable mass may also be present. Nonetheless, their existence or lack seems to have little bearing on the degree or presence of intussusception and the ensuing requirement for surgical exploration [21]. Laboratory findings may be unremarkable unless there is associated bowel ischemia, and physical examination may not reveal anything unless a palpable mass is present. In simpler terms, healthcare providers should be mindful of the potential presence of intussusception as a rare but serious hidden reason behind abdominal pain and vomiting in patients who have undergone RYGB surgery. This is particularly important in female patients who have experienced significant weight loss.

Management and Surgical Interventions

Clinical observations have revealed that intussusceptions occurring in patients who have undergone RYGB surgery may sometimes resolve on their own. However, in other instances, they can lead to a blockage in the intestine and may cause intestinal ischemia if not promptly addressed. As a result, urgent surgical intervention becomes a necessity. The clinical challenge lies in distinguishing cases that necessitate immediate surgical exploration from those that do not. Radiological results and assessing the patient's clinical complaints are the primary tools surgeons use to arrive at this decision. According to a study by Zaigham et al., when a patient has undergone an RYGB procedure, the best imaging examination to evaluate acute abdominal pain is an abdominal computed tomography (CT) scan. However, because this population frequently experiences unexplained chronic abdominal discomfort, it may be challenging to link the imaging results clinically, and additional research may call for a diagnostic laparoscopy. He concluded that an intussusception length of more than 100 mm on CT in patients with RYGB is a simple yet important indicator of a small intestinal blockage that would necessitate emergency surgery [19]. CT scans frequently show the well-known "target sign." It is composed of two layers: the inner (intussusceptum) and the outer (intussuscipiens), and within this mass is a densely packed area of fat. This area of fat accumulation shows the mesenteric fat that has invaded.

Distended bowel segments, with or without the presence of air-fluid levels, are possible additional findings. Furthermore, isolated distention of particular gastrointestinal tract segments, like the duodenum, gastric remnant, and biliopancreatic limb, may also occur. It is crucial to remember that these results may also be symptomatic of other problems, such as internal herniation, adhesions, or stenosis at the JJ site, and they are less specific for intussusception [21]. According to Bonouvrie et al., MRI may be the best imaging modality during pregnancy to reduce the amount of ionizing radiation the fetus is exposed to. It should also be done to make the diagnosis in pregnant patients [20]. Reduction of the intussusception, reduction with plication, resection of the afflicted bowel, and resection of the bowel with revision of the anastomosis are the four choices for managing RI, according to Machado et al. [17]. According to Bonouvrie et al., both manual reduction and surgical resection appear to be adequate treatment choices for intussusception during pregnancy [20]. Pregnant individuals with gravid uteri have also undergone laparoscopic procedures [23,24].

In cases where a part of the intestine gets blocked or becomes irreducible and is associated with bowel ischemia or tissue death (necrosis), the preferred treatment is surgical resection. Even though surgical correction of intussusception is linked to a considerable risk of wound infections, and the existing body of literature offering strong support for surgical resection over manual reduction is somewhat limited, the findings of the 2020 study conducted by Bonouvrie et al. underscore the importance of adopting a cautious approach. In cases where there is even a slight suspicion of irreversible ischemic damage, the preferred course of action should be surgical resection [20]. However, some authors have proposed that a less aggressive approach involving the reduction of the obstructed portion without resection may prove safe and offer potential advantages. These benefits could include preserving the length of the affected bowel, avoiding creating new surgical connections (anastomoses), and the associated risks of complications like leaks, bleeding, or the recurrence of intussusception. It is important to note that opting for more conservative measures might increase the chances of the condition recurring and the recurrence rates of the disease [2]. When applying a laparoscopic approach, managing the swollen bowel initially may pose some challenges, but with steady and gentle manipulation, the area of the swollen intussusception can be overcome. The complete resection of symptomatic lesions or complications in patients may determine the therapeutic approach.

Limitations

Given that most of the studies in our analysis primarily consisted of case reports and case series, it is essential to acknowledge the potential for selection bias in our findings. Furthermore, it is worth noting that there was a lack of standardized follow-up protocols employed for detecting intussusception in patients who had undergone primary RYGB. This absence of standardized procedures carries the risk of patients becoming lost to follow-up, which increases the likelihood of underestimating the actual incidence of intussusception following RYGB. Additional limitations in this review pertain to the limited number of cases available for analysis; this constraint diminishes the ability to provide the actual outcomes. Moreover, we should consider the incomplete nature of the data obtained from the studies, which further contributes to the potential uncertainties in our conclusions.

## Conclusions

Intussusception following RYGB surgery is a complex clinical condition with uncertain origins, diverse symptoms, and challenging diagnosis. Medical professionals must diligently monitor and follow up with RYGB patients, paying close attention to those experiencing abdominal pain and vomiting, as intussusception may be a concern. The management approach, whether conservative or surgical, depends on the clinical situation and the extent of ischemic damage. Due to the risk of recurrence, decisions regarding less aggressive treatment must be carefully considered. Further studies are essential to investigate long-term outcomes in BS patients and explore preventive measures for individuals at risk of intussusception after RYGB procedures, with the goal of reducing its incidence.
